# Interleukin-6 Trans-signaling: A Pathway With Therapeutic Potential for Diabetic Retinopathy

**DOI:** 10.3389/fphys.2021.689429

**Published:** 2021-05-19

**Authors:** Shruti Sharma

**Affiliations:** ^1^Center for Biotechnology & Genomic Medicine, Medical College of Georgia, Augusta University, Augusta, GA, United States; ^2^Department of Ophthalmology, Medical College of Georgia, Augusta University, Augusta, GA, United States; ^3^Culver Vision Discovery Institute, Medical College of Georgia, Augusta University, Augusta, GA, United States

**Keywords:** interleukin-6, IL-6 trans-signaling, sgp130Fc, inflammation, diabetic retinopathy

## New Therapies for Diabetic Retinopathy are an Urgent Unmet Need

Diabetic retinopathy (DR), a sight-threatening neurovasculopathy, is the leading cause of blindness in working-aged adults (Zhang et al., 2010; Hendrick et al., 2015). DR is characterized by pathologic vascular proliferation, oxidative damage, and inflammation within the retina (Tang and Kern, 2011; Antonetti et al., 2012; Klaassen et al., 2013). The progression of DR is highly correlated to the duration of diabetes (Fong et al., 2004). While restoring glycemic control and regulating other systemic factors are important for slowing DR development, limited therapeutic options are available once symptoms progress, and these are primarily aimed at treating late-stage disease (Fullerton et al., 2014; Do et al., 2015; Lee et al., 2015; Gardner and Sundstrom, 2017).

The number of DR patients is expected to increase over the coming decades. Currently, the only recommended treatments for advanced retinopathy are laser photocoagulation or anti-VEGF injections, but a substantial proportion of patients are resistant to these treatments. Laser photocoagulation can control pathological neovascularization, but it may lead to complications, such as impaired central vision, nocturnal diminution of vision, and blindness. The beneficial effects of anti-VEGF injections are usually transient, as the treatment does not promote tissue repair, and repeated injections increase the risk of intraocular infection. Furthermore, despite receiving anti-VEGF injections, a small proportion of patients with macular edema still show persistent disease (Lavine et al., 2017; Roy et al., 2017). Moreover, neither treatment targets early-stage disease. Another study examined the effect of candesartan, an angiotensin-II receptor antagonist, on patients with type 1 diabetes and found a moderate 18% reduction in incidence of retinopathy with no effect on the progression of existing retinopathy (Group and Chaturvedi, 2002). Therefore, new therapies to prevent retinal injury and enhance repair represent a critical unmet need.

## Interleukin-6 Trans-Signaling: A Potential Therapeutic Target for Diabetic Retinopathy

The pleiotropic cytokine, interleukin-6 (IL-6), is one of the major mediators of retinal vascular inflammation associated with DR (Shimizu et al., 2002; Funatsu et al., 2005; Mocan et al., 2006; Kawashima et al., 2007; Hou et al., 2008; Barnes et al., 2011; Koleva-Georgieva et al., 2011; Gustavsson et al., 2013; Koskela et al., 2013; Chen et al., 2016; Srividya et al., 2018; Valle et al., 2019). IL-6 signaling through its membrane-bound IL-6 receptor is known as “*classical signaling.”* Importantly, IL-6 signaling is also observed in cells that do not express the membrane-bound IL-6 receptor through a soluble IL-6 receptor (sIL-6R), known as “*trans-signaling”* (Barnes et al., 2011; Rose-John, 2012). There is increasing evidence in the literature suggesting that IL-6 classical signaling is anti-inflammatory, whereas trans-signaling induces the pro-inflammatory effects of IL-6 (Rabe et al., 2008; Ebihara et al., 2011; Fisher et al., 2011; Scheller et al., 2011; Wei et al., 2013). Trans-signaling has also been reported to have stronger effects than classical signaling (Reeh et al., 2019).

Recent advances in the field have led to the development of several therapeutic interventions targeting IL-6 signaling pathways, including anti-IL6 antibodies: siltuximab, sirukumab, olokizumab, and clazakizumab; anti-IL6R antibodies: tocilizumab, sarilumab, satralizumab, and vobarilizumab; and selective inhibitors of IL-6 trans-signaling only: sgp130Fc (olamkicept). Anti-IL6 and anti-IL6R therapeutic strategies globally block IL-6 signaling, essentially targeting both classical and trans-signaling pathways. Tocilizumab, an IL-6 receptor-inhibiting monoclonal antibody, is useful in the treatment of various autoimmune and inflammatory conditions, notably rheumatoid arthritis (Ohsugi and Kishimoto, 2008). However, this treatment was associated with negative side effects, such as liver toxicity and increases in triacylglycerol and cholesterol levels (Kawashiri et al., 2011).

Long-term hyperglycemia-mediated oxidative stress and inflammation lead to blood-retinal barrier (BRB) dysfunction and increased vascular permeability, allowing extravasation of plasma proteins into the interstitium (Frey and Antonetti, 2011; Klaassen et al., 2013). This dysfunction leads to edema, deposition of hard exudates in the retina, microaneurysms, and retinal hemorrhage (Cheung et al., 2010; Cunha-Vaz et al., 2011; Eshaq et al., 2017). BRB breakdown and subsequent macular edema are the main causes of blindness in DR (Antonetti et al., 1999; Joussen et al., 2007; Gardner et al., 2009; Klaassen et al., 2013; Sugimoto et al., 2013; Kita et al., 2015; Lee et al., 2015). IL-6 plays a significant role in initiating BRB breakdown in DR (Mesquida et al., 2019; Valle et al., 2019). Studies have shown that IL-6 signaling decreases barrier function in retinal endothelial cells and increases vascular leakage through downregulating tight junction proteins (Yun et al., 2017; Jo et al., 2019). IL-6 trans-signaling causes oxidative stress, inflammation, and endothelial barrier disruption in human retinal endothelial cells (Valle et al., 2019). Further, in a mouse model of early DR, inhibition of IL-6 trans-signaling significantly reduced diabetes-induced oxidative damage at the systemic level and in the retina (Robinson et al., 2020).

IL-6 also plays an important role in localized immune responses by mediating the recruitment of circulating leukocytes, attachment to the endothelium, and migration through the vascular wall (Romano et al., 1997; Rojas et al., 2010; Ebihara et al., 2011). Arrest and firm adhesion of leukocytes occur by their binding to endothelial cells using intercellular adhesion molecule 1 (ICAM-1) and vascular cell adhesion molecule 1 (VCAM-1). In diabetic patients, increased ICAM-1 expression in retinal vessels is correlated with an increase in migrating neutrophils (Noda et al., 2012). We previously found elevated levels of soluble ICAM-1 and VCAM-1 in patients with DR (Sharma et al., 2015) and increased ICAM-1 protein levels in human retinal endothelial cells after IL-6 trans-signaling activation (Valle et al., 2019). Numerous studies have demonstrated that IL-6 increases the expression of ICAM-1, VCAM-1, and selectins (Wung et al., 2005; Lin et al., 2013), but distinct roles of classical and trans-signaling have not been studied. Future studies delineating the relationship between IL-6 trans-signaling and leukocyte migration in the retinal vasculature will enhance our understanding of inflammation and BRB breakdown associated with DR.

## Molecular Tools for Delineating the Roles of IL-6 Classical and Trans-Signaling

### sgp130Fc

The soluble gp130 (sgp130) is a natural inhibitor of IL-6 trans-signaling (Wolf et al., 2016; Rose-John, 2017; Baran et al., 2018). The commercially available compound, sgp130Fc (soluble gp130Fc fused chimera), is an optimized fusion protein of the natural sgp130 and IgG1-Fc (Tenhumberg et al., 2008). sgp130Fc binds to IL-6 in complex with soluble IL-6R (IL-6/sIL-6R) and does not interfere with IL-6 alone or IL-6 bound to IL-6R on the cell surface. Therefore, sgp130Fc selectively inhibits IL-6 trans-signaling without disrupting IL-6 classical signaling via the membrane bound IL-6R. Compared to endogenous sgp130, sgp130Fc has been shown to possess 10 to 100 times greater ability for inhibiting IL-6 trans-signaling responses (Jostock et al., 2001). The use of this compound alongside existing global IL-6 inhibitors allows for a direct comparison of the therapeutic potential of global vs. selective trans-signaling inhibition.

### Hyper IL-6

Hyper-IL-6 is a fusion protein using a flexible peptide linker between soluble IL-6R and IL-6 to connect both molecules. Therefore, instead of a mixture of IL-6 and soluble IL-6R, hyper IL-6 can be used to stimulate IL-6 trans-signaling in cells. Also, hyper IL-6 is ~100 × more potent than the combination of IL-6/sIL-6R (Fischer et al., 1997; Jostock et al., 2001; Drucker et al., 2010). This compound is particularly useful for studies involving cells that express the membrane-bound IL-6 receptor, as a mixture of IL-6 and soluble IL-6R could theoretically activate both classical and trans-signaling. Hyper IL-6 allows for selective activation of IL-6 trans-signaling without any classical signaling activation.

### L-gp130

The transmission of the IL-6 signaling through the plasma membrane is mediated through glycoprotein 130 kDa (gp130). IL-6 receptor associates with the ubiquitously expressed protein gp130, initiating dimerization and intracellular signaling. L-gp130 is a designer protein in which the entire extracellular portion of gp130 is replaced by the leucine zipper of the Jun protein for constitutive dimerization and activation. Thus, L-gp130 protein can be used for permanent gp130 activation to mimic constitutive IL-6 signaling in cells (Stuhlmann-Laeisz et al., 2006).

### Transgenic Mice Overexpressing sgp130Fc

Transgenic mice that constitutively overexpress sgp130Fc are valuable resources to selectively block IL-6 trans-signaling *in vivo* (Rabe et al., 2008). Two types of transgenic mice are available for either central or peripheral expression of sgp130Fc. Peripheral sgp130Fc transgenic mice express sgp130Fc in the liver under the control of the phophoenol pyruvate-carboxykine (PEPCK) promotor for systemic release into the circulatory system (Rabe et al., 2008; Kraakman et al., 2015). The central sgp130Fc transgenic strain allows for inhibition of IL-6 trans-signaling in the central nervous system through sgp130Fc expression under control of a glial fibrillary acidic protein (GFAP) promotor (Campbell et al., 2014). Functionally, these models mimic intravenous (peripheral) or intravitreal (central) drug delivery, two common methods used in the treatment of ocular diseases.

## Concluding Remarks

Increasing evidence suggests that the IL-6 pathway plays a prominent role in the pathogenesis of DR. The complex IL-6 receptor system allows for multiple signaling modalities, including classical signaling and trans-signaling. Classical signaling is critical for the regenerative or anti-inflammatory activities of IL-6, while recent studies have demonstrated that IL-6 trans-signaling is primarily pro-inflammatory. In DR, IL-6 trans-signaling mediates barrier disruption in retinal endothelial cells, and blockade of this pathway maintained normal endothelial barrier function. Selective inhibition of IL-6 trans-signaling with sgp130Fc also suppressed ocular inflammation and oxidative stress in a mouse model of DR. These findings indicate that a pathway primarily driven by IL-6 + soluble IL-6R contributes to vascular inflammation in the diabetic retina. Therefore, inhibiting only the trans-signaling pathway of IL-6 will likely be therapeutically superior to a complete IL-6 blockade, because important physiologic functions of IL-6 classical signaling will remain intact. An emerging challenge is identifying means of targeting this inflammatory pathway, as well as determining which DR patients may benefit most from therapies blocking IL-6 trans-signaling. The selective inhibition of IL-6 trans-signaling using the sgp130Fc fusion protein is in clinical trials for the treatment of several inflammatory diseases (Rose-John, 2017) and may be repurposed in the future as an excellent target for DR therapy.

## Author Contributions

The author confirms being the sole contributor of this work and has approved it for publication.

## Conflict of Interest

The author declares that the research was conducted in the absence of any commercial or financial relationships that could be construed as a potential conflict of interest.
